# First report of *Neoechinorhynchus* (Acanthocephala: Neoechinorhynchidae) from marine fish of the eastern seaboard of Vietnam, with the description of six new species

**DOI:** 10.1051/parasite/2011181021

**Published:** 2011-02-15

**Authors:** O.M. Amin, N.V. Ha, D.N. Ha

**Affiliations:** 1 Institute of Parasitic Diseases 11445 E. Via Linda, # 2-419 Scottsdale Arizona 85259 USA; 2 Department of Parasitology, IEBR, Vietnam Academy of Science and Technology 18 Hoang Quoc Viet Cau Giay, Hanoi Vietnam; 3 Institute of Ecology and Biological Science Nghiado Cau Giay, Hanoi Vietnam

**Keywords:** Acanthocephala, *Neoechinorhynchus*, Neoechinorhynchidae, marine fish, Halong Bay, Vietnam, Acanthocephala, *Neoechinorhynchus*, Neoechinorhynchidae, poisson de mer, Baie d’Halong, Vietnam

## Abstract

The occurrence of acanthocephalans of the genus *Neoechinorhynchus* Stiles and Hassall, 1905 in Vietnamese waters is reported for the first time. Six new species are described from seven species of marine fish of the families Belonidae, Clupeidae, Megalopidae, Mugilidae, and Sciaenidae, collected in Halong Bay of the eastern seaboard of Vietnam in 2008 and 2009. These are *Neoechinorhynchus (Neoechinorhynchus) plaquensis* n. sp. characterized by dermal plaques covering the entire trunk; *Neoechinorhynchus manubriensis* n. sp. with very long anterior proboscis hooks having roots with prominent anterior manubria and very small and equal middle and posterior hooks, two pseudoretractors in the receptacle, simple vagina, and terminal gonopore; *Neoechinorhynchus pennahia* n. sp. with equal anterior and middle proboscis and somewhat smaller posterior hooks, and terminal female gonopore; *Neoechinorhynchus ampullata* with many giant nuclei in the body wall and lemnisci and a parareceptacle structure complex which includes pumping ampullas reported for the first time; *Neoechinorhynchus (Neoechinorhynchus) longinucleatus* n. sp. with very long giant nuclei in the Lemnisci, anteriorly twisted vagina, and subterminal female gonopore. *Neoechinorhynchus (Neoechinorhynchus) ascus* n. sp. is the second species of *Neoechinorhynchus* found with the parareceptacle structure/ampulla complex. *Neoechinorhynchus (Neoechinorhynchus) johnii* Yamaguti, 1929 of Bilqees, 1972 is not *N. johnii* because of proboscis armature and other discrepancies with the Yamaguti material. Notes on host distribution and feeding habits are also included.

## Abstract (fr)

La présence d’acanthocéphales du genre *Neoechinorhynchus* Stiles et Hassal, 1905 est signalée pour la première fois dans les eaux vietnamiennes. Six nouvelles espèces sont décrites chez sept poissons marins récoltés dans la Baie d’Halong sur la côte orientale du Vietnam en 2008 et 2009. Il s’agit de *Neoechinorhynchus (Neoechinorhynchus) plaquensis* n. sp., caractérisé par des plaques dermiques couvrant la totalité du tronc ; *Neoechinorhynchus manubriensis* n. sp., dont les crochets de la rangée antérieure sont très longs et présentent une racine pourvue d’un manubrium antérieur proéminant tandis que les crochets de la deuxième et de la troisième rangées sont petits et de taille équivalente, deux pseudo rétracteurs dans le réceptacle, un vagin simple et un gonopore terminal ; *Neoechinorhynchus pennahia* n. sp., avec les crochets des deux premières rangées de taille équivalente, une troisième rangée de petites épines et un gonopore femelle terminal ; *Neoechinorhynchus ampullata* avec de nombreux noyaux géants dans la paroi du corps et les lemnisques et un pararéceptacle, structure complexe, comprenant des ampoules signalées pour la première fois ; *Neoechinorhynchus (Neoechinorhynchus) longinucleatus* n. sp., avec de très longs noyaux géants dans les lemnisques, un vagin enroulé dans sa partie antérieure et un gonopore femelle subterminal. *Neoechinorhynchus (Neoechinorhynchus) ascus* n. sp. est la seconde espèce trouvée, du genre *Neoechinorhynchus*, présentant un complexe pararéceptacle/ampoules. *Neoechinorhynchus (Neoechinorhynchus) johnii* Yamaguti, 1929 de Bilqees, 1972 n’est pas *N. johnii* en raison de l’armature du proboscis ainsi que d’autres différences avec le matériel de Yamaguti. Des indications sur le spectre d’hôtes et la localisation dans le tube digestif de l’hôte sont également signalées.

## Introduction

Eleven acanthocephalan species from freshwater fish and other vertebrates were previously described in Vietnam by Amin & Ha (2008) and Amin *et al.* (2000, 2004, 2008 a, b, c). Eleven species of acanthocephalans were collected from marine fish off the eastern seaboard of Vietnam in 2008 and 2009. Of these, six species belong to *Neoechinorhynchus* Stiles and Hassall, 1905; all are new. No members of *Neoechinorhynchus* were ever reported anywhere in that country (Arthur & Te, 2006). The fact that the six reported species of *Neoechinorhynchus* are new only reflects the poor state of knowledge of the acanthocephalan fauna in Vietnam and adjacent countries with virtually no records of acanthocephalans.

## Materials and Methods

Of 45 species of marine fish netted at the Cat Ba Islands Tonkin Gulf, Halong Bay, North Vietnam (107°05’E, 20°45’N) during the spring of 2008 and 2009, 13 species were found infected with acanthocephalan parasites. Of these, seven fish species harbored acanthocephalans of the genus *Neoechinorhynchus* (Table I).

**Table I.** Prevalence and intensity of infection of marine fish from Halong Bay, Vietnam, with acanthocephalans of the genus *Neoechinorhynchus.*

	Fish		Acanthocephalans		
Name	Length range (mean) cm	Infect/exam.	Collected	Range	Mean
Belonidae					
Strongylura strongylura	34–37 (35)	2/2 (100 %)	12	4–8	6.00
Clupeidae					
Clupanodon thrissa	15–17 (16)	2/24 (8.3 %)	3	1–2	0.12
Megalopidae					
Megalops cyprinoids	9–11 (10)	4/4 (100 %)	4	1	1.00
Mugilidae					
Valamugil seheli	10–24 (16)	10/46 (22 %)	19	1–5	0.41
Sciaenidae					
Johnius carouna	10–16 (14)	1/16 (6 %)	5	5	0.31
Nibea albiflora	19–24 (21)	2/14 (14 %)	3	1–2	0.21
Pennahia argentata	11–31 (21)	1/22 (4 %)	2	2	0.09

Upon collection fish were measured and photographed then brought to the laboratory for examination. Worms were placed in water for 2-5 hours or until fully extended then fixed in 70 % ethanol. Worms were punctured with a fine needle and subsequently stained in Mayer’s acid carmine, destained in 4 % hydrochloric acid in 70 % ethanol, dehydrated in ascending concentrations of ethanol (24 hours each), and cleared in graduated (increasing) concentrations of terpineol in 100 % ethanol to 100 % terpineol, then 50 % terpineol in 50 % Canada balsam (24 hours each). Whole worms were mounted in Canada balsam.

Measurements are in micrometers, unless otherwise stated. Range values are followed by the mean in parentheses. Length measurements are given before the width; the latter refers to maximum width. Trunk length does not include the neck, proboscis, or bursa. Eggs refer only to fully developed eggs usually removed from the body cavity. Specimens were deposited in the University of Nebraska’s State Museum’s Harold W. Manter Laboratory (HWML) collection in Lincoln, Nebraska.

## Results and Discussion

The six taxa described in this work are all new species of *Neoechinorhynchus* collected from seven species of marine fishes in Halong Bay off the eastern seaboard of Vietnam in 2008 and 2009 (Table I). This is the first report of any species of *Neoechinorhynchus* anywhere in Vietnam.

### *Neoechinorhynchus (Neoechinorhynchus) Plaquensis* n. sp. (Figs 1-7)

Two worms (one male, one female) of three worms collected from two of 24 examined Chinese gizzard shad, *Clupanodon thrissa* (Linnaeus) (Clupeidae) in January 2008 were available for study. Both specimens were mature with sperm and eggs. The host, *C. thrissa* is a pelagic tropical marine fish commonly found in coastal waters, but tolerates brakish and freshwater, in the Northwest Pacific of China and Vietnam, and feeds on finfish and phytoplankton (Whitehead, 1985).

**Figs 1-7.**
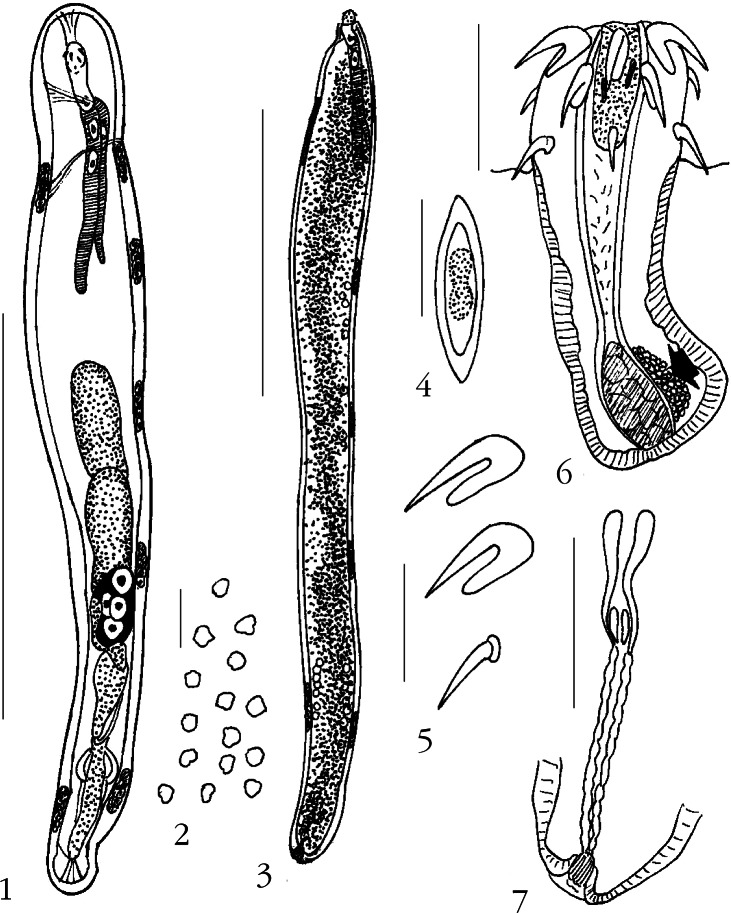
*Neoechinorhynchus (Neoechinorhynchus) plaquensis* n. sp. 1. Holotype male. 2. Dermal plaques from the trunk of the holotype male. 3. Allotype female. 4. Egg. 5. Proboscis hooks. 6. Proboscis and receptacle of allotype female showing cell pouch (arrow) at its posterior end. 7. Female reproductive system. Measurement bars: 1, 3: 3 mm; 2: 10 μm; 4: 20 μm; 5: 50 μm; 6: 125 μm; 7: 500 μm.

#### • Description

General: Neoechinorhynchidae. With characters of the genus *Neoechinorhynchus* and subgenus *Neoechinorhynchus* as described by Amin (2002). Trunk cylindrical, widest anterior to middle, elongate, straight. Minimal sexual dimorphism except in body size. Body wall with five dorsal and two ventral giant hypodermal nuclei (Figs 1, 3) and many very small sparsely and randomly distributed, amorphous plaques throughout (Fig. 2). Proboscis with apical organ having two elongate giant nuclei, almost rectangular, slightly longer than wide, with flat indentation at anterior corners (Fig. 6). Proboscis hooks decrease slightly in size posteriorly and of similar length in both sexes: 57, 48, 45 in females and 57, 50, 44 in males. Hooks in anterior two circles very close together and with similar prominent simple roots directed posteriorly. Posterior hooks begin near posterior tip of middle hooks and with knob-like basal disks, not true roots (Fig. 5). Neck short. Proboscis receptacle slightly more than twice as long as proboscis with large drop-shaped cephalic ganglion at base and prominent dorsal cell pouch with very thin barely visible membrane at its posterior end adjacent to cephalic ganglion (Fig. 6, arrow). Lemnisci subequal, cylindrical, of medium length, with one and two giant nuclei in shorter and longer lemniscus, respectively (Fig. 1). Gonopore terminal in male and near terminal in female (Figs 1, 3, 7).

Male (holotype): trunk 6.35 mm long by 0.82 mm wide. Proboscis retracted but not invaginated, 130 long by 112 wide. Proboscis receptacle 337 long by 82 wide.

Lemnisci not reaching anterior testis. Longer lemniscus 1,414 long by 135 wide; shorter lemniscus 1,123 long by 125 wide. Reproductive system in posterior half of trunk. Testes contiguous oblong; anterior testis 832 long by 364 wide, posterior testis 884 long by 354 wide. Cement gland contiguous with posterior testis, elliptoid, with four prominent giant nuclei, 541 long by 270 wide. Common sperm duct 416 long by 83 wide. Seminal vesicle wider anteriorly, 551 long by 218 wide. Saefftigen’s pouch slanted, 603 long by 114 wide. Genital terminalia funnel-shaped with prominent muscular rim.

Female (allotype): trunk 8.62 mm long by 0.80 wide. Proboscis 125 long by 117 wide. Proboscis receptacle 267 long by 82 wide. Longer lemniscus 1,508 long by 125 wide; shorter lemniscus 1,404 long by 125 wide. Reproductive system reduced with undulating uterine wall, 884 long (10 % of trunk length), one unremarkable terminal vaginal sphincter, and simple uterine bell lacking pouches or cells. Eggs fusiform with concentric shells, 27-30 long by 10-12 wide (Fig. 4). Gonopore near terminal (Fig. 7).

#### • Taxonomic summary

Type host: Chinese gizzard shad, *Clupanodon thrissa* (Linnaeus) (Clupeidae).

Type locality: Halong bay at Cat Ba Island, Vietnam (107°05’E, 20°45’N).

Site of infection: intestine.

Type specimens: HWML collection no. 49210 (holotype male and allotype female on same slide).

Etymology: the new species is named for the many small plaques found throughout the body wall of both sexes.

#### • Remarks

*Neoechinorhynchus (N.) plaquensis* is the only species of *Neoechinorhynchus* that has dermal plaques covering the whole trunk as well as the proboscis receptacle cellular pouch adjacent to the cerebral ganglion. Only occasional specimens of *Neoechinorhynchus (Hebesomo) tenellus* (Van Cleave, 1913) Van Cleave, 1919 had cuticular plaques at anterior and posterior ends of the trunk (Amin & Muzzall, 2009) while specimens of other genera, *e.g.*, *Sclerocollum rubrimaris* Schmidt and Paperna, 1978 are characterized with dermal plaques. The simplified female reproductive system lacking uterine bell cells and the proportion of hook sizes from anterior to posterior with the distance between hooks in the various circles are also only characteristic of this species. Its proboscis armor is somewhat similar to that of *Neoechinorhynchus roonwali* Datta and Soota, 1961 from *Orienus* sp. in Afghanistan in hook size. In *N. roonwali*, however, the lemnisci are 2.0-3.8 mm long with 3-6 nuclei each and the hypodermal nuclei in the body wall are 9-10 dorsally and two ventrally (Datta & Soota, 1961). The marine *Neoechinorhynchus ningalooensis* Pichelin and Cribb, 2001 collected from parrotfish, *Scarus* spp. in Western Australia also bears some superficial similarities to *N. plaquensis* n. sp. However, *N. ningalooensis* has markedly larger trunk, proboscis, proboscis hooks, proboscis receptacle, and testes, and its male reproductive system is displaced much more posteriorly (Pichelin & Cribb, 2004).

### *Neoechinorhynchus Manubriensis* n. sp. (Figs 8-14)

Of the 10 worms collected from Caroun croaker, *Johnius carouna* (Cuvier) (Sciaenidae) (five worms) in December 2008, the white flower croaker, *Nibea albiflora* (Richardson) (Sciaenidae) (three worms) in July 2008, and the silver croaker, *Pennahia argentata* (Houttuyen) (Sciaenidae) (two worms), in July 2008, six specimens (four males and two females) of this new species were studied. All three species of croakers are benthopelagic fish found in coastal waters, with *J. carouna* occasionally entering estuaries and mangrove swamps, of the Indo-Pacific (*J. carouna*) and or the Northwest Pacific (*N. albiflora* and *P. argentata*). They all feed on Zoobenthos, crustaceans, prawns, other invertebrates and finfish (Sasaki, 2001; Trewavas, 1977).

**Figs 8-14.**
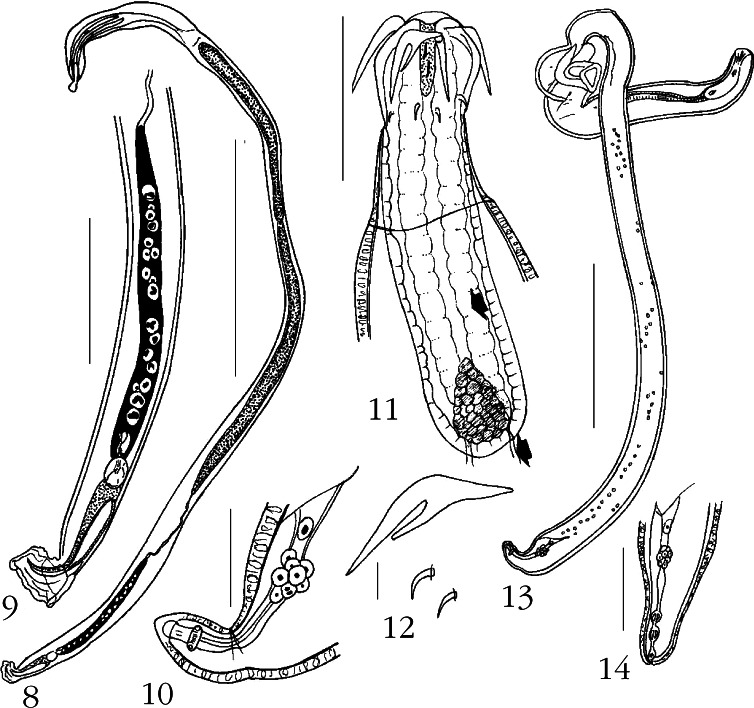
*Neoechinorhynchus manubriensis* n. sp. 8. Holotype male. 9. Detail of the posterior end of the holotype male. 10. Reproductive system of allotype female in Fig. 13. 11. Proboscis and receptacle of holotype male. Note the pseudo-retractors and their posterior filaments (arrows). 12. Proboscis hooks of allotype female. 13. Allotype female. 14. Reproductive system of a juvenile female. Measurement bars: 8: 4 mm; 9: 1 mm; 10, 11: 200 μm; 12: 30 μm; 13: 2 mm; 14: 70 μm.

#### • Description

General: Neoechinorhynchidae. With characters of the genus *Neoechinorhynchus* as described by Amin (2002). Trunk elongate cylindrical and slender throughout but posterior end of female abruptly attenuates (Figs 10, 13). Giant hypodermal nuclei not readily observed. No sexual dimorphism in proboscis armature. Proboscis slightly longer than wide with flat anterior and anteriolateral sides and small elongate apical organ with two elongate giant nuclei (Fig. 11). Anterior proboscis hooks 98-112 long with posteriorly directed roots having prominent anterio-lateral manubria. Middle and posterior hooks small, rootless, equal in size, 25-27 long (Fig. 12). Neck prominent, as long as proboscis but wider at base. Proboscis receptacle about three times as long as proboscis, with a prominent basal triangular cephalic ganglion and two lobulated undulating pseudo-retractors connected with posterior filaments (Fig. 11). Lemnisci nearly equal, elongate, with one and two large giant nuclei in shorter and longer lemniscus, respectively (Fig. 8). Gonopore terminal in both sexes (Figs 9, 10).

Males (based on four adults): trunk 5.87-15.00 (10.2) mm long by 0.25-0.42 (0.34) mm wide. Proboscis 120-137 (128) long by 105-127 (116) wide. Hook length from anterior 98-112 (102), 25-27 (26), 25-27 (26). Neck 135 long by 146 wide at base. Proboscis receptacle 406-426 (416) long by 130-135 (132) wide. Lemnisci 1,248-1,875 (1,618) long by 83-94 (88) wide. Reproductive system in posterior 80 % of trunk. Testes tubular, fill body cavity laterally; anterior testis 750-3,250 (2,095) long by 125-260 (184) wide, posterior testis twice as long, 1,450-5,275 (2,579) long by 125-250 (177) wide. Cement gland long, tubular, a distance away from posterior testis, 645-2,200 (1,237) long by 83-175 (122) wide, with 1 column of 16-20 (18) giant nuclei. Cement reservoir contiguous with cement gland, 156-228 (192) long by 104-156 (130) wide, sperm vesicle 728 long by 156 wide, and bursa 416 long by 260 wide in longest specimen (holotype). Saefftigen’s pouch 384-541 (462) long by 67-145 (106) wide (Figs 8, 9).

Females (based on one juvenile and one adult): trunk 3.80-8.75 (6.27) mm long by 0.27-0.32 mm wide. Proboscis hooks from anterior 98-102 (100), 27, 27. Proboscis receptacle 312 long by 104-146 (125) wide. Lemnisci 988-1,300 (1,116) long by 62 wide. Reproductive system 130 long in juvenile and 500 long in adult, 3.4 % and 5.7 % of trunk length, respectively. Gonopore terminal in both juvenile (Fig. 14) and adult (Fig. 10) but vagina is more developed in adult specimen. Uterine bell attached to body wall, with large cells and one large giant nucleus and slanted anterior end in both specimens. Posterior end with terminal gonopore and attenuates more drastically in adult (Figs 10, 13) than in juvenile specimen (Fig. 14). Eggs not developed.

#### • Taxonomic summary

Type host: caroun croaker, *Johnius carouna* (Cuvier) (Sciaenidae).

Other hosts: flower croaker, *Nibea albiflor*a (Richardson); silver croaker, *Pennahia argentata* (Houttuyen) (Sciaenidae).

Type locality: Halong Bay at Cat Ba Island, Vietnam (107°05’E, 20°45’N).

Type specimens: HWML Collection no. 49211 (holotype male and paratype from *Johnius carouna*), no. 49212 (allotype female and paratype from *Pennahia argentata*).

Etymology: the new species is named for the prominent antero-lateral manubrium of the root of its anterior proboscis hook.

#### • Remarks

The new species belongs in a group of five long slender acanthocephalans of Asian marine fish having similar proboscis armature characterized by very long anterior hooks and very small middle and posterior hooks (Table II). The proboscis hooks of *N. manubriensis* are most similar to those of *N. johnii* sensu Yamaguti (1939) in size. The new species is distinguished from the latter (described by Yamaguti, 1939 only from females but by Gupta and Jain, 1983 from both sexes) by having the anterior hook with prominent manubrium, a triangular cephalic ganglion, two pseudo-retractors in the receptacle, equal lemnisci, simple vagina, and terminal gonopore. *Neoechinorhynchus johnii* of Bilqees (1972) is clearly another species; it differs from *N. johnii* of Yamaguti (1939) in having proboscis hooks that gradually decrease in size posteriorly. The anterior testis of the males described by Bilqees (1972) is oval in contrast with the posterior tubular testis.

**Table II.** Comparison of key taxonomic characteristics between *Neoechinorhynchus manubriensis* n. sp. and related species from marine fish in Asia.

Characters	*N. manubriensis* n. sp. This paper	*N. (N.) johnii* Yamaguti, 1939 Gupta & Jain, 1983	“*N. johnii*” Bilqees, 1972	*N. (N.) tylosuri* Yamaguti, 1939	*N. topseyi* Podder, 1937 Gupta & Jain, 1983
Distribution	Halong Bay, Vietnam	East China Sea, Arabian Sea	Karachi Coast, Pakistan	Russia, Japan, China, Iran, Black Sea	West coast of India, Arabian Sea
Host	Jonius carouna	Jonius goma	Protonibea diacanthus	Enchelycore	Filimanus
	Nibea albiflora Pennahia argentata	Protonibea diacanthus	Qonius goma)	schismatorhynchus	heptadactylus
Prob. Hook L ♂♂	98–112, 25–27, 25–27	89–93, 20–24, 20–24	100, 30–40, 17–20	63–69, 30, 30–35	81–87, 26–28, 21–24
(ant, mid, post) ♀♀	98–102, 27, 27	90–100, 21–24, 21–24	80–90, 50–60, 19–20	75, 35, 35	85–93, 28–30, 24–28
Ant. hook root	With large manubrium	No manubrium	No manubrium	No manubrium	No manubrium
Pseudo-retractors	Present	Absent	Absent	Absent	Absent
Cephalic ganglion	Triangular	Round	Round	Oval	Ovoid/ameboid
Lemnisci	Equal	Unequal	Subequal	Unequal	Subequal
Testes	Tubular	Elongate	Ant. oval, post. tubular	Elliptical to cylindrical	Ovoid/elongate
Cement gland	Long, 16–20 nuclei	Long, 12–13 nuclei	Long, many nuclei	Long, 22 nuclei	Rounded/elongate
Vagina	Simple	Complex bulb with folds	Complex bulb with folds	Simple, twisted	Simple
Female gonopore	Terminal	Subterminal	Subterminal	Subterminal	Subterminal

Our new species also differs from *Neoechinorhynchus tylosuri* Yamaguti, 1939 in practically all characters listed in table II except for the type of cement gland and vagina (Yamaguti, 1939). It is similar to *Neoechinorhynchus topseyi* Podder, 1937 in the size proboscis hooks and type of vagina but differs in all other characters listed in Table II (Podder, 1937).

Two other species, *Neoechinorhynchus (N.) africanus* Troncy, 1969 from the freshwater fish *Citharinus distichoides* Pellegrin in Chad and *Neoechinorhynchus (N.) dorsovaginatus* Amin and Christison, 2005 from *Argyrosomus japonicus* (Temminck and Schlegel) off the South African coast also bear similarities to the new species. In *N. africanus*, however, the proboscis hooks decrease in size posteriorly from 90 to 50 to 30 long, the neck is long, 60, and is distinctly separated from the trunk and the eggs have a spiny belt (Troncy, 1969). In *N. dorsovaginatus*, the trunk is shorter and very thick-walled with anterodorsal hump, the proboscis is very large, anterior hooks are smaller, 70-83, and the neck is much longer and houses the receptacle, the cement gland with 30 nuclei, and the female gonopore and vaginal sphincter are dorso-subterminal (Amin & Christison, 2005). Neither of these two species has anterior hook roots or receptacle retractors similar to those of *N. manubriensis*.

### *Neoechinorhynchus Pennahia* n. sp. (Figs 15-18)

Only one female was collected from the only infected individual of *Pennahia argentata* (Houttuyen) (Sciaenidae) in July, 2008. That same infected host also harbored one specimen of *N. manubriensis*. The silver croaker, *P. argentata*, is a benthopelagic temperate fish that inhabits the coastal waters of the Norwest Pacific and feeds on zooplankton, various invertebrates and small fin fishes (Trewavas, 1977).

**Figs 15-18.**
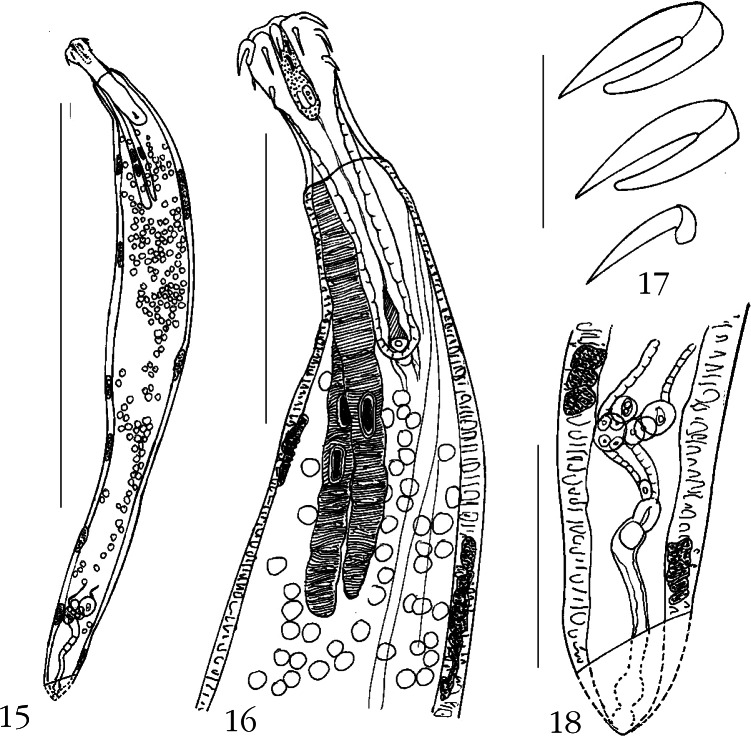
*Neoechinorhynchus pennahia* n. sp. 15. Holotype female. 16. Anterior end of the holotype showing the relationship between the proboscis, receptacle, and lemnisci. 17. Proboscis hooks. 18. Reproductive system of holotype indicating the reconstructed posterior tip. Measurement bars: 15: 2 mm; 16: 500 μm; 17: 55 μm; 18: 250 μm.

#### • Description

General: Neoechinorhynchidae. With character of the genus *Neoechinorhynchus* as described by Amin (2002). Female trunk small straight cylindrical and somewhat wider in anterior half, with six ventral and three dorsal giant hypodermal nuclei (Fig. 15). Proboscis ovoid with long apical organ having two elongate giant nuclei anteriorly and two posterior cells (Fig. 16). Anterior and middle hooks equal and relatively longer than posterior hooks. All hooks with simple posteriorly directed roots; roots of posterior hooks abbreviated (Fig. 17). Neck prominent, longer than proboscis, with parallel sides, slightly wider only at base. Proboscis receptacle about four times as long as proboscis with elongate triangular cephalic ganglion 87 long by 25 wide at base. Lemnisci finger-like, slender, subequal, considerably longer than receptacle (Fig. 16), with two and one giant nuclei in longer and shorter lemniscus respectively.

Holotype female: trunk 3.12 mm long by 0.45 mm wide. Proboscis 132 long by 120 wide. Proboscis hooks from anterior 55, 55, 37-42. Neck 142 long by 125 wide at base. Long leminiscus 832 long by 62 wide, short lemniscus 728 long by 55 wide. Uterine bell very short and wide with prominent but few cells. Gonopore terminal. Posterior tip broke off during processing but illustration completed from original sketch (Fig. 18).

#### • Taxonomic summary

Type host: silver croaker *Pennahia argentata* (Houttuyen) (Sciaenidae).

Type locality: Halong Bay at Cat Ba Island, Vietnam (107°05’E, 20°45’N).

Site of infection: intestine.

Type specimen: HWML Collection no. 49213 (holotype female).

Etymology: the new species is named for the generic name of the host.

#### • Remarks

*Neoechinorhynchus pennahia* n. sp. is the only species of *Neoechinorhynchus* characterized by a combination of its proboscis armature, long neck, subequal lemnisci, and terminal female gonopore. Only the freshwater *Neoechinorhynchus (Hebesoma) idahoensis* Amin and Heckmann, 1992 from *Catostomus columbianus* in Idaho, USA, has similar proboscis armature with hook lengths of 48-61 (56), 48-61 (54), and 40-51 (46) from anterior in females. *Neoechinorhynchus idahoensis*, however, has distinctly subterminal female gonopore, a notched proboscis receptacle posteriorly, and lemnisci considerably more different in length (Amin & Heckmann, 1992). The new species is also distinguished from two other species of *Neoechinorhynchus* also with proboscis hooks in anterior and middle circles of equal length, *e.g.*, *Neoechinorhynchus notemigoμni* Dechtiar, 1967 and *Neoechinorhynchus crassus* Van Cleave, 1919. The anterior hooks of these two species are, however, shorter than 40 μm or longer than 90 μm, among other differences, respectively (Dechtiar, 1967; Van Cleave, 1919).

### *Neoechinorhynchus Ampullata* n. sp. (Figs 19-23)

Four worms (two males, two females) were collected from four individual Indo-Pacific tarpons, *Megalops cyprinoids* (Broussonet) (Megalopidae) in January, 2008. The host is a tropical Indo-Pacific benthopelagic amphidromous (brakish, freshwater, marine) fish that was captured in marine waters of the Cat Ba islands of Halong Bay. It is common from the Red Sea and Natal, South Africa to the South China Sea, Taiwan Strait and East China Sea and feeds primarily on zoobenthos and nekton (Coates, 1987). One of the two female worms collected was too contorted out of shape to be of any use. The two males specimens had sperm and the remaining female specimen (allotype) had only ovarian balls and no eggs.

**Figs 19-23.**
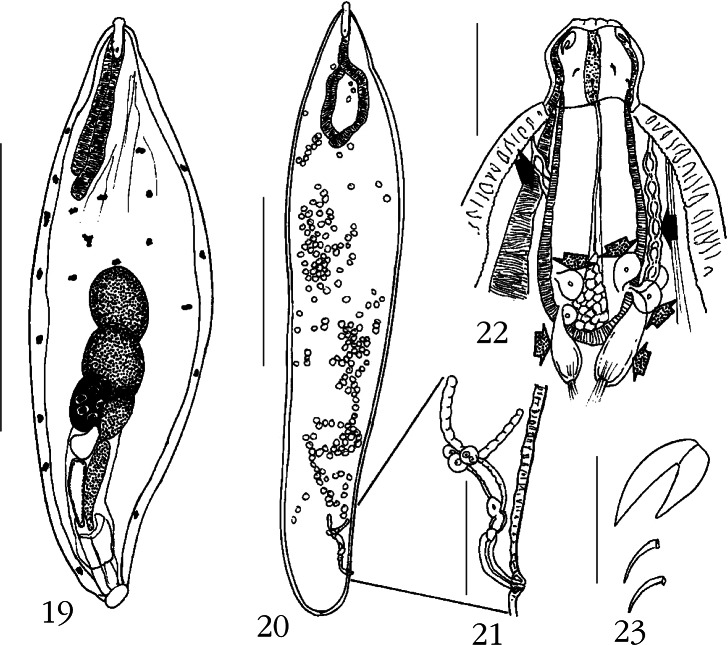
*Neoechinorhynchus ampullata* n. sp. 19. Holotype male. 20. Allotype female. 21. Reproductive system of allotype female. 22. Proboscis and receptacle of allotype female showing parareceptacle structures (solid arrows) and ampullas (granular arrows) of the parareceptacle complex. 23. Proboscis hooks of allotype female. Measurement bars: 19, 20: 3 mm; 21: 700 μm; 22: 250 μm; 23: 50 μm.

#### • Description

General: Neoechinorhynchidae. With characters of the genus *Neoechinorhynchus* as described by Amin (2002). Trunk relatively small, fusiform, especially in males but may be wider more anteriorly in some females (Figs 19, 20) with marked sexual dimorphism. Body wall with eight dorsal, four ventral, and additional lateral (nine on latero-anterior side on one male) giant nuclei (Fig. 19). Proboscis as long as wide in males but wider than long in female, with long apical organ having two elongate anterior nuclei. Proboscis hooks in middle and posterior circles equal in size and about half as long as anterior hooks but considerably more slender. Only anterior hooks with sizable simple spoon-like roots directed posteriorly; middle and posterior hooks rootless (Fig. 23). Neck short. Proboscis receptacle with one ventral and one dorsal parareceptacle structures attached anteriorly to body wall and insert posteriorly into receptacle (Fig. 22, solid arrows) and associated posteriorly with blind bladder-like ampullas (Fig. 22, granular arrows) by proximal insertion into proboscis receptacle near its posterior end. Posterior end of ampullas held by bands of muscular fibers extending into body cavity. Lemnisci cylindrical, plump, subequal, widest posteriorly, with four giant nuclei in each.

Males (based on two adults): trunk 5.17-5.82 (5.50) mm long by 1.25-1.87 (1.56) mm wide at middle. Proboscis 105 long by 105 wide. Proboscis hooks 32-37 (35), 17, 17 long from anterior. Proboscis receptacle 302 long by 115-117 (116) wide. Longer lemniscus 1.87-2.44 (2.16) mm long by 0.16-0.21 (0.19) mm wide at base; shorter lemniscus 1.61-2.17 (1.89) mm long by 0.16-0.21 (0.19) mm wide at base. Reproductive system in posterior half of trunk. Anterior testis 741-850 (795) long by 437-550 (493) wide. Posterior testis relatively shorter, 478-825 (651) long by 468-575 (521) wide. Cement gland contiguous with posterior testis; with 6 giant nuclei, 520-625 (572) long by 374-425 (400) wide. Cement reservoir partially overlapping cement gland, 187-332 (259) long by 208-239 (223) wide. Common sperm duct just posterior to posterior testis, 530 long by 343 wide. Sperm vesicle 801-874 (837) long by 229 wide, just posterior to common sperm duct and adjacent but slightly anterior to Saefftigen’s pouch. Gonopore terminal.

Female (allotype): trunk 10.77 mm long by 2.10 mm wide (Fig. 20). Proboscis 110 long by 147 wide. Proboscis hooks 44, 20, 20 long from anterior. Proboscis receptacle 510 long by 177 wide (Fig. 22). Longer lemniscus 2.96 mm long by 0.23 mm wide at base; shorter lemniscus 2.34 mm long by 0.23 mm wide at base. Reproductive system 1.404 mm long (13 % of trunk length) with reduced number of uterine bell cells. Gonopore distinctly subterminal, 811 distance from posterior end of trunk (Fig. 21).

#### • Taxonomic summary

Type host: Indo-Pacific tarpon, *Megalops cyprinoids* (Broussonet) (Megalopidae).

Type locality: Halong Bay at Cat Ba Island, Vietnam (107°05’E, 20°45’N).

Site of infection: intestine.

Type of specimens: HWML Collection no. 49214 (holotype and paratype males on one slide), no. 49215 (allotype female and paratype on one slide).

Etymology: the new species is named for the bladder-like ampullla associated with the parareceptacle structure characteristic of this species.

#### • Remarks

*Neoechinorhynchus ampullata* is the first species of the genus that is reported to posess bladder-like ampullas that insert through the wall of the proboscis receptacle near its posterior end. The few ampullas in each specimen were translucent and appear to contain clear liquid. These ampullas may be accessory structures (pumps) associated with the paired dorsal and ventral parareceptacle structures (PRS) that, also insert into the receptacle wall nearby. The PRS has been suggested to regulate the hydrostatic pressure in the receptacle to facilitate the retraction and eversion of the proboscis in some eoacanthocephalans with weak single-walled proboscis receptacle (Amin *et al.*, 2007). One other species described at the end of this article is also observed to have similar parareceptacle structure/ampulla complex. The PRS alone, without the ampulla, has been previously reported in three other species of Eoacanthocephalans, *Neoechinorhynchus (Neoechinorhynchus) qatarensis* Amin, Saoud, and Alkuwari, 2002; *Neoechinorhynchus (Neoechinorhynchus) golvani* Salgado- Maldonado, 1978 (Neoechinorhynchidae); and *Acanthogyrus (Acanthosentis) parareceptaclis* Amin, 2005 (Quadrigyridae) (see Amin *et al.*, 2007).

The new species is further distinguished from related species of *Neoechinorhynchus* as follows. It is most closely similar to *Neoechinorhynchus (Hebesoma) manasbalensis* Kaw, 1951 known from freshwater fish in Kashmir in body shape, similar number of giant nuclei and similar reproductive structures in both sexes. In *N. manasbalensis*, however, the lemnisci are nearly equal and much shorter and the proboscis hooks are considerably longer 46-60, 30-38, 28-34 long from anterior (Kaw, 1951).

The shape of the trunk, reproductive structure, lemnisci, and position of female gonopore are also similar to those of *Neoechinorhynchus (Neoechinorhynchus) paraguayensis* Machado, 1959 from freshwater fishes in Brazil and Paraguay, and *Neoechinorhynchus pterodoridis* Thatcher, 1981 from freshwater fishes in Brazil. Both species, however, have few giant nuclei (five and one in body wall) and much longer anterior hooks: 82-100 and 100-130 long in males and females of *N. paraguayensis*, and 142-145 long and 130-148 long in males and females of *N. pterodoridis* (Machado, 1959; Thatcher, 1981). Only two additional species of *Neoechinorhynchus* similary have many giant nuclei, *Neoechinorhynchus (Neoechinorhynchus) chilkaensis* Podder, 1937 from *Barbus* in brakish waters of Ropar, India, and *Neoechinorhynchus devdevi* Datta, 1936 in freshwater fish of Kashmir Valley. Both species, however have cylindrical trunks and much longer anterior hooks: 65-70 and 68-78 long in male and female *N. chilkaensis* (Podder, 1937) and 90 long in both males and females of *N. devdevi* (Datta, 1936).

### *Neoechinorhynchus (Neoechinorhynchus) Longinucleatus* n. sp. (Figs 24-31)

Twelve specimens (five males, seven females) of this new species were collected in May, 2009 from two spottail needlefish, *Strongylura strongylura* (Van Hasselt) (Belonidae) (Table I). Males included four sexually mature specimens with sperm and one juvenile. Females included two gravid specimens with eggs, four specimens with ovarian balls only, and one juvenile. The spottail needlefish is a pelagic-neritic marine and brakish water fish found in the Indo-West Pacific including the Persian Gulf eastward along the coast of Pakistan, India, and Sri Lanka then extending to southern China, the Philippines, and northern Australia (Collette, 1984).

**Figs 24-31.**
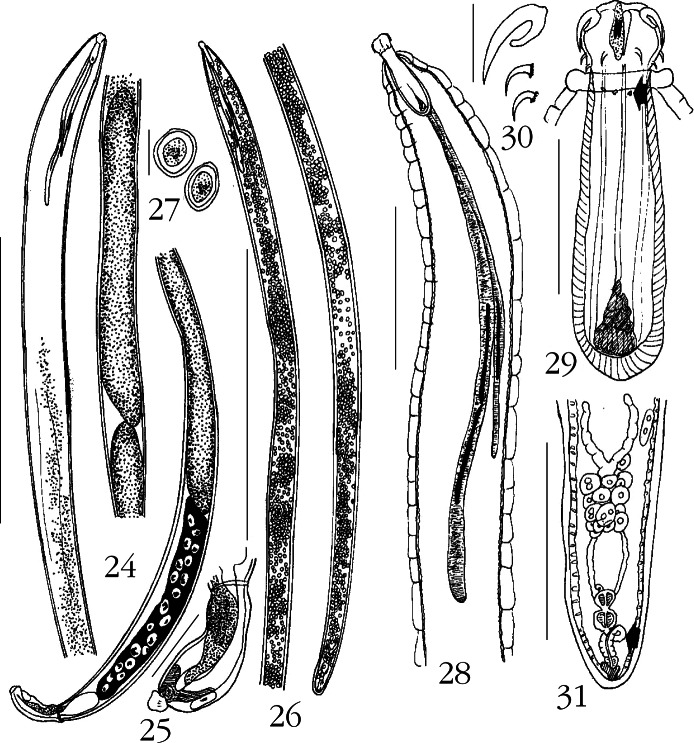
*Neoechinorhynchus (Neoechinorhynchus) longnucleatus* n. sp. 24. Holotype male; anterior testis often much closer to lemnisci. 25. Bursa of holotype male. 26. Allotype female. 27. Egg. 28 Anterior end of paratype male. 29. Proboscis and receptacle of the same paratype male in Fig. 28 showing the apical structure, anterior trunk collar, dermal plaques (arrow), and the two types of nuclei of the cephalic ganglion. 30. Proboscis hooks of a paratype male. 31. The reproductive system of the allotype female showing the multiple uterine bell cells and the twisted anterior part of the vagina (arrow). Measurement bars: 24: 5 mm; 25, 31: 500 μm; 26: 4 mm; 27: 30 μm; 28: 1 mm; 29: 250 μm; 30: 50 μm.

#### • Description

General: Neoechinorhynchidae. With characters of the genus *Neoechinorhynchus* and the subgenus *Neoechinorhynchus* as described by Amin (2002). Trunk long, slender, cylindrical, with parallel sides throughout (Figs 24, 26) giant hypodermal nuclei occasionally visible as cresent-shaped structures of moderate or more often small size. Anterior tip of trunk with 4-7 loosely arranged amoeboid or amorphous cuticular plaques (arrow) and prominent collar (Fig. 29). No sexual dimorphism in all shared structures. Proboscis as long as wide with posteriorly blunt apical organ containing two elongate giant nuclei (Fig. 29) Middle and posterior hooks equal, rootless, much shorter than anterior hooks. Roots of anterior hooks simple, posteriorly directed, crescent-shaped, about half as long as hooks (Fig. 30). Proboscis receptacle about four times as long as proboscis, with large triangular cephalic ganglion at base of two distinct types of nuclei (Fig. 29). Lemnisci long, slender, distinctly unequal, with very long and thin giant nuclei, 2 and 1 in longer and shorter lemniscus, respectively (Figs 24, 26, 28).

Males (based on four adults with sperm) [measurements of one immature male follow in brackets]: trunk 23.75-42.50 (31.42) mm long by 0.62-1.25 (0.88) mm wide [8.87 × 0.17]. Proboscis 102-130 (118) long by 110-120 (114) wide [100 × 100]. Proboscis hooks length from anterior 60-67 (64), 22-32 (27), 22-30 (26) [57-60, 25, 27]. Proboscis receptacle 415-457 (440) long by 110-146 (133) wide [337 × 100]. Cephalic ganglion 105-125 (112) long by 37-62 (50) wide. Lemnisci may extend beyond anterior testis posteriorly. Longer lemniscus 3.29-4.57 (3.86) mm long by 0.09-0.24 (0.14) mm wide. Shorter lemniscus 1.56-2.63 (2.18) mm long by 0.05-0.09 (0.07) mm wide (Fig. 28). Reproductive system in posterior 80 % of trunk, terminating at posterior tip of bursa. Testes long, rod-shaped, contiguous, as wide as inner diameter of body cavity. Anterior testis 5.00-8.30 (6.65) mm long by 0.50-0.55 (0.52) mm wide. Posterior testis somewhat longer, 6.37-9.25 (7.81) mm long by 0.55-0.75 (0.65) mm wide. Cement gland 3.50-6.87 (4.73) mm long by 0.37-0.75 (0.54) mm wide, with 15-24 (19) prominent round giant nuclei. Cement reservoir contiguous with posterior end of cement gland 509-750 (645) long by 250-300 (270) wide [208 X 93] (Fig. 24). Sperm vesicle 760 long by 187 wide and Safftigen’s pouch 905-1250 (1077) long by 104-250 (177) wide [416 X 602] completely inside extended bursa, 915 long by 395 wide [322 × 114] (Fig. 25).

Females (based on two gravid specimens and four with ovarian balls only) [measurements of one immature specimen in brackets]: trunk 9.57-44.57 (23.41) mm long by 0.29-0.95 (0.61) mm wide [5.75 × 0.24]. Proboscis 107-130 (120) long by 112-125 (118) wide [112 × 112]. Length of proboscis hooks from anterior 60-70 (65), 25-27 (26), 25-27 (26) [67-70, 27, 27]. Proboscis receptacle 374-480 (422) long by 125-146 (137) [364 × 125]. Cephalic ganglion 110-137 (122) long by 35-77 (60) wide [105 X 75]. Longer lemniscus 2.63-4.81 (3.56) mm long by 0.07-0.14 (0.10) wide [2.55 × 0.05]. Shorter lemniscus 1.17-2.46 (1.96) mm long by 0.05-0.07 (0.06) mm wide [1.58 × 0.04]. Reproductive system 572-780 (700) long (5.3 % of trunk length) [500, 9 %] with many uterine bell cells. Vagina twisting anteriorly and vulva terminal in juvenile and in adults of various sizes (Fig. 31). Eggs ovoid to round with concentric shells, 26-32 (29) long by 17-25 (22) wide (Fig. 27).

#### • Taxonomic summary

Type host: spottail needlefish, *Strongylura strongylura* (Van Hasselt) (Belonidae).

Type locality: Halong Bay at Cat Ba Island, Vietnam (107°05’E, 20°45’N).

Site of infection: intestine.

Type specimens: HWML Collection no. 49216 (holotype male and paratype female on same slide), no. 49217 (allotype female and paratype male on same slide).

Etymology: the new species is named for the very long giant nuclei of the lemnisci.

#### • Remarks

The new species is a member of the six species group including long and slender worms known from marine fish in Asia. Of the five other species (Table II), *N. longinucleatus* is closest to *N. tylosuri* in size of proboscis armature and shape of male and female reproductive systems. In *N. tylosuri*, however, the female gonopore is subterminal, the lemnisci and anterior testis are widely separated, the eggs are more elongate, 30-33 × 15-18, the cephalic ganglion is oval, the proboscis is larger, and the giant nuclei of the cement gland are elongate (Yamaguti, 1939). All other members of this group of worms (Table II) have considerably longer anterior hooks; *N. manubriensis* and “*N. johnii*” of Bilqees (1972) have equal lemnisci, *N. johnii* has complex vagina with folds and subterminal female gonopore (Yamaguti, 1939), and *N. topseyi* has oval testes, rounded cement gland, and subterminal female gonopore (Podder, 1937) (Table II).

### *Neoechinorhynchus (Neoechinorhynchus) Ascus* n. sp. (Figs 32-38)

Of 46 specimens of bluespot mullets, *Valamugil seheli* (Forsskål) (Mugilidae) examined, 10 were infected with 19 acanthocephalans (11 males, eight females) belonging in this new species. All worms were studied. The host, *V. seheli* is a reef-associated catadromous tropical fish of the Indo-Pacific distributed from the Red Sea south to South Africa and east to the Hawaiian islands, north to southern Japan, and south to New Caledonia. It inhabits coastal waters but enters estuaries and rivers where it feeds on microalgae, filamentous algae, formas, and detritus associated with sand and mud (Harrison & Senou, 1997; Myers, 1991).

**Figs 32-38.**
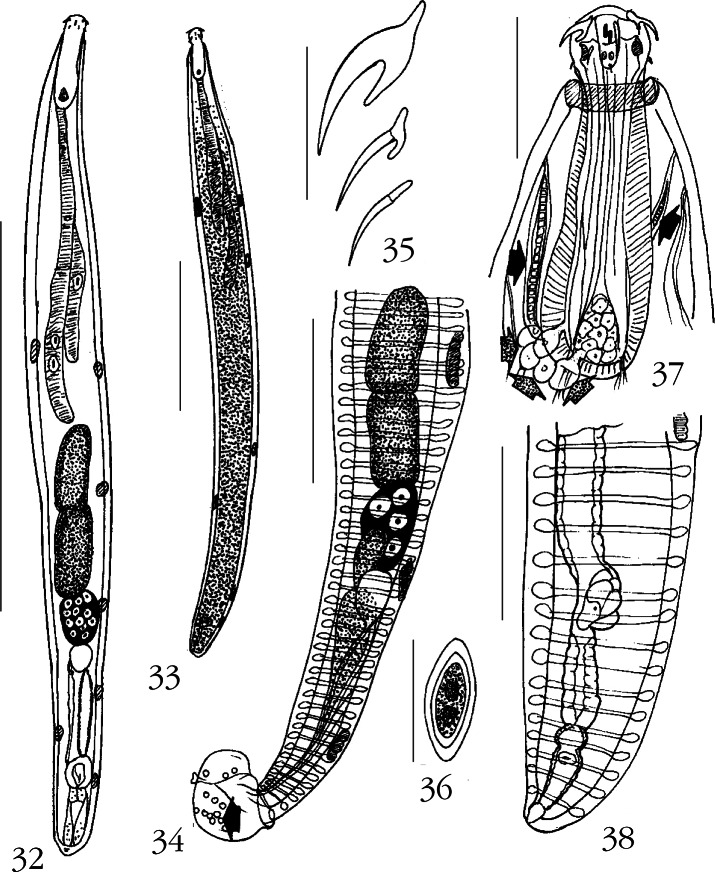
*Neoechinorhynchus (Neoechinorhynchus) ascus* n. sp. 32. Young paratype male; cement gland with 12 giant nuclei, reproductive system with only Saefftigen’s pouch well developed and mid-trunk not as wide as in mature adults. 33. Gravid paratype female. 34. Reproductive system of mature adult holotype male. Note sensory cells on bursa (arrow). 35. Proboscis hooks of a paratype male. 36. Egg. 37. The proboscis and receptacle of allotype female showing the parareceptacle structures (solid arrows) and ampullas (granular arrows) of the parareceptacle complex. 38. Reproductive system of a paratype female. Measurement bars: 32, 33: 2 mm; 34: 1 mm; 35: 75 μm; 36: 35 μm; 37: 300 μm; 38: 400 μm.

#### • Description

General: Neoechinorhynchidae: With characters of the genus *Neoechinorhynchus* and the subgenus *Neoechinorhynchus* as described by Amin (2002). Trunk cylindrical, straight, elongate, and slender with males wider at middle (Figs 32, 33), and with horizontal lacunar canals (Figs 34, 38). Trunk with anterior collar and 4-7 dorsal and 1-2 ventral hypodermal nuclei (Figs 32, 33). Medium sized females about twice as long as small males with sexual dimorphism in size of proboscis receptacle lemnisci, and cephalic ganglion. Proboscis globular, dome shaped anteriorly, wider than long, with apical organ that may extend to posterior end of proboscis and that has two prominent and vertically elongate giant nuclei anteriorly and two round nuclei posteriorly. Anterior proboscis is bold, free of armature (Fig. 37). Hooks progressively decrease in size posteriorly; all rooted. Anterior and middle hooks with similar prominent crescent-shaped roots and anterior manubria. Posterior hooks smallest with prominent rod-shaped root along same axis as hook (Fig. 35). Proboscis receptacle three and five times as long as proboscis in males and females, respectively, and with ventral and dorsal para-receptacle structures (Fig. 37, solid arrows) and posterior accessory ampulla-like sacs (Fig. 37, granular arrows). Cephalic ganglion triangular prominent, at posterior end of receptacle. Lemnisci long, cylindrical, subequal, occasionally extending past anterior testis in males and corresponding distance in females, with two and one giant nuclei in longer and shorter lemniscus, respectively (Figs 32, 33).

Males (based on 11 adults with sperm): trunk widest in mature specimens, 4.00-6.92 (5.49) mm long by 0.40-1.05 (0.76) mm wide at middle with 4-7 (usually four or five) dorsal and 1-2 (usually two) giant hypodermal nuclei. Proboscis 117-142 (131) long by 130-155 (143) wide. Proboscis hooks from anterior 70-87 (78), 45-52 (48), 35-42 (41) long. Proboscis receptacle 437-562 (502) long by 146-177 (162) wide. Cephalic ganglion 92-137 (110) long by 50-75 (65) wide at base. Longer lemniscus 1674-2881 (2247) long by 90-135 (112) wide; shorter lemniscus 1612-2860 (2086) long by 83-125 (110) wide. Reproductive system in posterior half of trunk and occasionally extending to posterior end of bursa. Testes and cement gland oblong, contiguous, and usually of similar size. Anterior testis 437-832 (617) long by 198-447 (321) wide; posterior testis 541-790 (626) long by 208-447 (300) wide. Cement gland 281-801 (606) long by 198-385 (311) wide, usually with four prominent giant nuclei but occasionally with eight or 12 nuclei. Contiguous cement reservoir 146-364 (260) long by 114-250 (211) wide, with two lateral cement ducts extending to posterior end of trunk. Common sperm duct prominent in mature adults, overlapping posterior half of cement gland. Sperm vesicle centro-posterior to cement reservoir but mostly between two cement ducts posteriorly, 468-572 (514) long by 177-260 wide anteriorly (Fig. 34). Saefftigen’s pouch only discernible in immature adults (Fig. 32). Bursa 312-780 (471) long by 229-468 (324) wide with clusters of sensory cells (Fig. 34, solid arrow).

Females (based on eight adults, two with eggs): trunk 8.27-14.87 (10.25) mm long by 0.67-1.45 (0.87) mm wide with 5 or 6 dorsal and 2 ventral giant nuclei. Proboscis 112-145 (126) long by 122-162 (141) wide. Proboscis hooks from anterior 65-92 (74), 40-52 (47), 35-47 (40). Proboscis receptacle 572-634 (603) long by 135-229 (178) wide. Cephalic ganglion 107-150 (127) long by 57-82 (66) wide at base. Longer lemniscus 2756-4160 (3312) long by 97-187 (141) wide; shorter lemniscus 2735-3900 (3137) long by 97-160 (135) wide. Reproductive system 687-1227 (869) long (6 % of trunk length in longest worms, and 11 % of trunk length in smaller worms: mean 8 %) (Fig. 38). Eggs fusiform with concentric shells (Fig. 36). Gonopore subterminal.

#### • Taxonomic summary

Type host: bluespot mullet, *Valamugil seheli* (Forsskål) (Mugilidae).

Type locality: Halong Bay at Cat Ba Island, Vietnam (107°05’E, 20°45’N).

Site of infection: intestine.

Type specimens: HWML Collection no. 49218 (holotype and paratype males on one slide, no. 49219 (allotype female and other paratype females on one slide). Etymology: the new species is named for the sac-like ampulla associated with the parareceptacle structure characteristic of this species.

#### • Remarks

*Neoechinorhynchus ascus* is the second species of the genus *Neoechinorhynchus* with a sac-like ampullas inserting into the receptacle wall at its posterior end. These bladder-like sacs appear like pumps associated with the parareceptacle structure that also inserts into the receptacle wall nearby, and that has been suggested to regulate the hydrostatic pressure in the receptacle to facilitate the retraction and the eversion of the proboscis in some eoacanthocephalans with weak single-walled proboscis receptacle (Amin *et al.*, 2007). This complex of ampulla and parareceptacle structure has been reported only in one other species of Eoacanthocephala, *N. ampullata* described earlier in this paper. The parareceptacle structure alone, without the ampulla, has been previously reported in three other species of eoacanthocephalans, *N. (N.) qatarensis*, *N. (N.) golvani*, and *Acanthogyrus (A.) parareceptaclis* (see Amin *et al.*, 2007). The new species is readily distinguishable from *N. ampullata*, which has a fusiform trunk, many giant nuclei in the hypodermis (eight dorsal, four ventral) and the lemnisci (4,3), and considerably smaller proboscis hooks (32-44, 17-20, 17-20 from anterior) with the anterior hooks lacking manubria. It is also distinguished from all other species of *Neoechinorhynchus* by having all proboscis hooks rooted with anterior and middle hooks having similar roots with anterior manubria.

Two other species, *Neoechinorhynchus (Neoechinorhynchus) chilkaensis* Podder, 1937 from India, and *Neoechinorhynchus karachiensis* Bilqees, 1972 from Pakistan superficially resemble *N. ascus* n. sp. In *N. chilkaensis*, however, the proboscis hooks are smaller (50-60, 23-32, 19 in males and 60-75, 26-38, 24-27 in females from anterior), anterior hook root without manubria, and cement gland with 6 giant nuclei. In *N. karachiensis*, the proboscis hooks are smaller (50-60, 39-40, 10-11 in males and 60-70, 40-50, 9-11 in females from anterior), the anterior and middle hooks root have no manubria, and the eggs are smaller 10-20 X 5-10. None of these two species has parareceptacle structure/ ampulla complex.

## Discussion

Available literature on the acanthocephalans from Vietnamese waters did not show any indication of the possible presence of any species of the genus *Neoechinorhynchus*. These reports by Amin & Ha (2008), Amin *et al.* (2000, 2004, 2008a, b, c) and Arthur and Te (2006) from freshwater fish and other vertebrates were all negative for acanthocephalans of this genus. No marine fish parasites were examined. In this very first exploration of marine fish parasites of the Vietnamese coast, a unique collection of six new species of acanthocephalans of the genus *Neoechinorhynchus* was discovered while none was found in freshwater fish in Vietnam even in the Red River which empties in the same Gulf of Tonkin. In North America, by comparison, practically all species of *Neoechinorhynchus* are known from freshwater fish and some from turtles (Amin, 2002). This reverse disparity of the distribution of marine vs. freshwater forms is unexplainable at the present time. We do not know if any one or more of these six new species are indigenous and of restricted distribution in the Gulf of Tonkin or if they are more readily dispersed in their host that often have wider distributional ranges.

The presence of *Neoechinorhynchus* species with unusual features is noted with great interest. In *N. plaquensis*, dermal plaques covering the whole trunk are observed for the first time. The only two species with parareceptacle structure/ampulla complex, *N. ampullata* and *N. ascus* were found in two of the six species studied from this locality while none was reported in the 88 species studied by Amin (2002) from all over the world and the few more species described since. The recovery of two new species, *N. manubriensis* and *N. longinucleatus*, belonging to a larger group of *Neoechinorhynchus* spp. from Asian marine fish characterized by long slender bodies and large anterior proboscis hooks (Table II) is also noted with interest.

The diversity of these species of *Neoechinorhynchus* was noted only from Halong Bay. The Bay is bordered on the south and southeast by the gulf of Tonkin, on the north by China and on the West and southwest by Cat Ba Island. The Bay area has a unique geological history beginning about 500 million years ago before the sea became shallow. The surrounding sea is now only 6-10 m deep except along old river channels, the result of marine transgression caused by the sinking of the underlying limestone plateau. In all, there are 1,929 islands and islets. According to the IEBR study, there are six aquatic sub-ecosystems containing rich reserves of plankton, mulloscs, sand worms, echinorderms, fish (400 species), bluegreen algae, seaweeds, coral, snails, crabs, sponge, and crustaceans (nine species) (anonymous, 2003; Knccn & Hnu, 1997; Tran *et al.*, 2004; Waltham, 1998).

## References

[R1] Amin O.M. Revision of *Neoechinorhynchus* Stiles and Hassall, 1905 (Acanthocephalan: Neoechinorhynchidae) with keys to 88 species in two subgenera. Systematic Parasitology, 2002, 53, 1–18 12378129 10.1023/a:1019953421835

[R2] Amin O.M. & Christison K.W. *Neoechinorhynchus (Neoechinorhynchus) dorsovaginatus* n. sp. (Acanthocephala: Neoechinorhynchidae) from the dusky kob *Agyrosomus japonicas* (Sciaenidae) on the southern coast of South Africa. Systematic Parasitology, 2005, 61, 173–179 16025206 10.1007/s11230-005-3130-1

[R3] Amin O.M. & Ha N.V. On a new acanthocephalan family and a new order from birds in Vietnam. Journal of Parasitology, 2008, 94, 1305–1310 18576806 10.1645/GE-1473.1

[R4] Amin O.M., Ha N.V. & Heckmann R.A. New and already known acanthocephalans from amphibians and reptiles in Vietnam, with keys to species of *Pseudoacanthocephalus* Petrockenko, 1956 (Echinorhynchidae) and *Sphaerechinorhynchus* Johnston and Deland, 1929 (Plagiorhynchidae). Journal of Parasitology, 2008a, 94, 181–189 18372639 10.1645/GE-1288.1

[R5] Amin O.M., Ha N.V. & Heckmann R.A. New and already known acanthocephalans mostly from mammals in Vietnam, with descriptions of two new genera and species in Archiacanthocephala. Journal of Parasitology, 2008b, 94, 194–201 18372641 10.1645/GE-1394.1

[R6] Amin O.M., Ha N.V. & Heckmann R.A. Four new species of acanthocephalans from birds in Vietnam. Comparative Parasitology, 2008c, 75, 200–214

[R7] Amin O.M. & Heckmann R.A. Description and pathology of *Neoechinorhynchus idahoensis* n. sp. (Acanthocephala: Neoechinorhynchidae) in *Catostomus columbianus* from Idaho. Journal of Parasitology, 1992, 78, 34–39 1738067

[R8] Amin O.M., Heckmann R.A. & Ha N.V. On the immature stages of *Pallisentis (Pallisentis) celatus* (Acanthocephala: Quadrigyridae) from occasional fish hosts in Vietnam. The Raffles Bulletin of Zoology, 2004, 52, 593–598

[R9] Amin O.M., Heckmann R.A. & Standing M.D. The structural functional relationship of the para-receptacle structure in Acanthocephala. Comparative Parasitology, 2007, 74, 383–387

[R10] Amin O.M., Heckmann R.A., Ha N.V., Luc P.V. & Doanh P.N. Revision of the genus *Pallisentis* (Acanthocephala: Quadrigyridae) with the erection of three new subgenera, the description of *Pallisentis (Brevitritospinus) vietnamensis* subgen. et sp. n., a key to species of *Pallisentis*, and the erection of a new quadrigyrid genus, *Pararaosentis* gen. n. Comparative Parasitology, 2000, 67, 40–50

[R11] Amin O.M. & Muzzall P.M. Redescription of *Neoechinorhynchus tenellus* (Acanthocephala: Neoechinorhynchidae) from *Esox lucius* (Esocidae) and *Sander vitreus* (Percidae), among other percid and centrarchid fish, in Michigan USA. Comparative Parasitology, 2009, 76, 44–50

[R12] Anonymous Ha Long Bay Management Department of Ha Long City (HLBMD). State of the World Heritage in the Asia-Pacific Region. Vietnam. Ha Long Bay Report to the UNESCO World Heritage Committee, Paris, France, 2003

[R13] Arthur J.R., Te B.Q. Checklist of parasites of fishes of Vietnam. FAO fisheries Technical Paper 369/2, 2006, 133 pp.

[R14] Bilqees F.M. Description of two Acanthocephala, including a new species *Neoechinorhynchus karachiensis* (Neoechinorhynchidea: Neoechinorhynchidae), from marine fishes of Karachi. Sind University Research Journal, 1972, 6, 93–100

[R15] Coates D. Observations on the biology of tarpon, *Megalops cyprinoids* (Broussonet) (Pisces: Megalopidae), in the Sepik River, northern Papua New Guinea. Australian Journal of Marine and Freshwater Research, 1987, 38, 529–535

[R16] Collette B.B. *In*: FAO species identification sheets for fishery purposes. Western Indian Ocean (Fishing area 51) Fischer W. & Bianchi G., (eds), FAO, Rome, 1984, vol. 1

[R17] Datta M.N. Scientific results of the Yale North India expedition. Biological Report no. 20, 1936, 38, 211–229

[R18] Datta M.N. & Soota T.D. A new species of Acanthocephala *Neoechinorhynchus roonwali* from Afghanastan, and notes on *Pomphorhynchus kashmiriensis* Kaw. Records of the Indian Museum, 1961, 59, 35–39

[R19] Dechtiar A. *Neoechinorhynchus notemigoni* n. sp. (Acanthocephala: Neoechinorhynchidae) from golden shiner of Lake Ontario. Canadian Journal of Zoology, 1967, 45, 155–159 10.1139/z71-073 5106577

[R20] Gupta N.K. & Jain M. On three already known species of the genus *Neoechinorhynchus* Hamann, 1892 (Acanthocephala) from fish in the Arabian Sea. Acat Parasitologica Polonica, 1983, 18, 407–416

[R21] Harrison I., Senouh J. Order Mugiliformes Mullets, *in*: FAO species identification guide for fishery purposes. The living marine resources of Western Central Pacific, Vol. 4. Bony fishes part 2 (Mugillidae to Carangidae) Carpenter K.E. & Niem V.H. (Eds), FAO, Rome, 1997, 2069–2108

[R22] Kaw B.L. Studies in Helminthology: Helminth parasites of Kashmir. Part 2. Acanthocephala. Indian Journal of Helminthology, 1951, 3, 117–132

[R23] KNCCN, IEBR, HNU Ecosystem and biodiversity of Cat Ba National park and Ha Long Bay, Vietnam. Seoul: The Korean national Council for Conservation of Nature, the Institute of Ecology and Biological Resources, Hanoi, and Hanoi National University, 1997

[R24] Machado D.A. Uma nova espécie de gěnero *Neoechinorhynchus* Hamann, 1892, Parasite de “peixe-martin” do Paraguai (Neoechinorhynchidae: Archiacanthocephala). Revista Brasileirade Biologia, 1959, 19, 379–381

[R25] Myers R.F. Micronesion reef fishes, Second edn. Coral Graphics, Barrigada, Guam, 1991, 298 pp.

[R26] Pichelin S & Cribb T.H. *Neoechinorhynchus ningalooenensis* sp. nov. (Acanthocephala: Neoechinorhynchidae) from *Scarus ghobban* and *S. psittacus* (Scaridae) from Western Australia. Transations of the Royal Society of South Australia, 2001, 125, 51–55

[R27] Podder T.N. A new species of Acanthocephala, *N. topseyi* n. sp. from a Culcutta Fish, *Polynemus heptadactylus* (Cuv. Et Val.). Parasitology, 1937, 29, 365–369

[R28] Sasaki K. Sciaenidae. Sciaenidae. Croakers (drums) p 3117-3174, *in*: FAO species identification guide for fishery purposes. The living marine resources of the Western Central Pacific. Bony fishes part 3 (Menidae to Pomacentridae) Carpenter K.E. & Niem V.H. (eds), Rome, FAO, 2001, 2791–3380

[R29] Thatcher V.E. *Neoechinorhynchus pterodoridis* n. sp. (Acanthocephala: Neoechinorhynchidae) do bacu liso (*Pterodoras granulosus*) da Amazônia Brasileira. Acta Amazonica, 1981, 11, 445–448

[R30] Tran V.T., Tran S.T., Waltham T., Li S.A. & Lal & H.A. The Ha Long Bay World Heritage: Outstanding Geological values, National Committee for ICCP, Vietnam, 2004

[R31] Trewavas E. The sciaenid fishes (croakers or drum) of the Indo-west Pacific. Transactions of the Zoological Society of London, 1977, 33, 253–541

[R32] Troncy P.M. Contribution à l’étude des helminthes d’Afrique, principalment du Tchad. 1. Acanthocephales. Bulletin du Muséum National d’Histoire Naturelle, 1970, 41, 1487–1511

[R33] van Cleave H.J. Acanthocephala from the Illinois River with descriptions of species and a synopsis of the family Neoechinorhynchidae. Illinois Natural History Survey Bulletin, 1919, 13, 237–257 (plates 22-28)

[R34] Waltham T. Limestone Karst of Ha Long Bay, Vietnam: An assessment of the Karst geomorphology of the world heritage site, Report to Nottingham Trent University, 1998

[R35] Whitehead P.J.P. FAO species catalogue. Vol. 7. Clupeoid fishes of the world (suborder Clupeioidei). An annotated and illustrated catalogue of the herrings, sardines, pilchards, sprats, shads, anchovies and wolf-herrings. Part 1. Chirocentridae, Clupeidae and Pristigasteridae FAO fish. Synop, 1985, 125, pp 1–303

[R36] Yamaguti S. Studies on the helminth fauna of Japan. Part 29. Acanthocephala, II. Transactions of the Japanese Journal of Zoology, 1939, 13, 317–351

